# Sexual Trajectories of Non-heteronormative Identities in South African Pentecostalism: A Scoping Review Protocol

**DOI:** 10.12688/f1000research.171581.1

**Published:** 2025-11-04

**Authors:** Mashudu Edward Muthivhi, Muimeleli Munyadziwa

**Affiliations:** 1 Human Sciences, University of Venda, Thohoyandou, Limpopo, 0950, South Africa; 2 Public Health, University of Venda, Thohoyandou, Limpopo, 0950, South Africa

**Keywords:** LGBTQ+; Non-heteronormative; Pentecostalism, Practical theology; sexuality; South Africa

## Abstract

Non-heteronormative persons within South African Pentecostal communities experience unique challenges that arise from the intersection of sexual orientation and religious belief. Although the South African constitutional framework ostensibly grants rights and protections to sexually diverse individuals, Pentecostal expressions of Christianity largely continue to uphold heteronormative understandings, rendering sexual diversity undesirable and invisible. This scoping review paper aims to systematically map the literature on the experiences, theological discourses, and pastoral care of non-heteronormative persons in South African Pentecostalism, utilising Arksey and O’Malley’s framework. By analysing, collating, and summarising the existing body of work, the review aims to identify categories of evidence and information gaps to facilitate the possibility of further developing inclusive theological and pastoral practices and paradigms within/pastoral care within South African Pentecostalism. Ultimately, the findings will guide researchers, policymakers, and faith communities in devising interventions and responses to foster acceptance, inclusion, and well-being.

## Introduction

The intersection of sexual orientation and religious conviction presents complex challenges for individuals navigating non-heteronormative identities within faith-based communities. ^1– 3^ In South Africa, despite progressive constitutional protections for LGBTQ+ individuals, including legal recognition of same-sex civil unions ^4^ religious institutions, particularly Pentecostal churches, often maintain heteronormative frameworks that marginalise sexual and gender diversity. ^5– 7^ Pentecostalism, characterised by its emphasis on literal biblical interpretation, personal spiritual experiences, and moral rectitude, frequently frames non-heteronormative sexualities as morally deviant, perpetuating exclusionary practices and psychological distress among adherents. ^1– 3^

The societal and theological marginalisation of LGBTQ+ individuals in Pentecostal settings reflects broader tensions between evolving social values and religious orthodoxy. ^8, 9^ South Africa’s multicultural and postcolonial context further complicates these dynamics, as traditional African cultural norms and Pentecostal moral imperatives converge to reinforce heteronormativity. ^10^ Non-heteronormative individuals often face the dual pressure of adhering to community-sanctioned religious and cultural expectations while striving for personal authenticity, creating a persistent dilemma with significant psychosocial and spiritual consequences. ^11, 12^

Pentecostal theological discourse frequently invokes heteronormative hermeneutics, such as interpretations of Genesis 19 (Sodom and Gomorrah), Leviticus 18:22, Leviticus 20:13, Romans 1:26-27, 1 Corinthians 6:9, and 1 Timothy 1:10, to validate exclusionary practices. ^12– 14^ These readings, however, are increasingly contested by queer theological scholarship, which emphasises inclusivity, spiritual affirmation, and the recognition of diverse sexual identities as integral to the human experience. ^15, 16^ The disconnect between rigid heteronormative interpretations and lived experiences of LGBTQ+ individuals underscores the need for nuanced, contextually informed analyses of how Pentecostalism in South Africa engages or fails to engage with non-heteronormative trajectories. ^2, 5^

Although existing research has examined queer theology, pastoral care, and LGBTQ+ experiences in faith contexts globally, there is a notable paucity of literature systematically mapping these phenomena within the South African Pentecostal milieu. ^15, 17, 18^ A scoping review is therefore warranted to consolidate current knowledge, identify gaps, and provide a foundation for future research, theological reflection, and inclusive pastoral practice. ^13, 19, 20^ By synthesising empirical and theoretical literature, this review will illuminate the ways Pentecostal communities influence the expression, negotiation, and acceptance of non-heteronormative identities, thereby informing both scholarly discourse and practical interventions that promote inclusivity and spiritual well-being. ^9, 12, 20, 21^

### Aim

To systematically map literature on the experiences, theological interpretations, and pastoral care of non-heteronormative individuals within South African Pentecostalism.

### Objectives

1. To identify existing literature on non-heteronormative sexual identities within South African Pentecostal communities.
2. To explore theological and pastoral responses to non-heteronormative sexualities.
3. To examine the psychosocial and spiritual experiences of LGBTQ+ individuals in Pentecostal contexts.
4. To highlight gaps in knowledge to inform future research, theology, and pastoral practice.

### Scoping review questions

This scoping review seeks to systematically map the literature on non-heteronormative sexual identities within South African Pentecostal communities. Guided by the PEO framework.

• **Population (P):** Non-heteronormative individuals (LGBTQ+) within South African Pentecostal communities
• **Exposure (E):** Experiences within Pentecostal contexts, including theological interpretations and pastoral care
• **Outcome (O):** Psychosocial, spiritual, and pastoral outcomes, including inclusion, acceptance, and well-being

The primary research question is:

• What evidence exists on the experiences, theological interpretations, and pastoral care of non-heteronormative individuals in South African Pentecostalism, and how do these factors influence their inclusion, acceptance, and well-being?

***Sub questions***

1. What evidence is available on the lived experiences of non-heteronormative individuals within South African Pentecostal communities?
2. In what ways do Pentecostal theological interpretations shape perceptions and attitudes toward sexual diversity?
3. What pastoral care approaches or interventions are documented to support non-heteronormative individuals in Pentecostal settings?
4. What gaps exist in the current literature that warrant further research on non-heteronormative identities in South African Pentecostalism?

### Research design and methods

***Study design***

This study will employ a scoping review design guided by the Arksey and O’Malley framework. ^22^ Scoping reviews are appropriate for mapping broad topics, identifying key concepts, and highlighting gaps in the literature.

***Search strategy***

A comprehensive and systematic search strategy will be employed to identify relevant literature on non-heteronormative sexual identities within South African Pentecostal contexts. The search will be conducted across multiple electronic databases, including Web of Science, PubMed, SABINET, JSTOR, and Google Scholar, to ensure broad coverage of both health, social science, and theological literature.

Key search terms will include:

• “LGBTQ”
• “Queer”
• “Sexual minorities”
• “Pentecostalism”
• “Pentecostal churches”
• “South Africa”
• “Southern Africa”
• “Sexuality”
• “Identity”
• “Spirituality”
• “Pastoral care”

Boolean operators (AND, OR) will combine keywords, and reference lists of included articles will be screened for additional sources.

***Selection of sources***

***Inclusion criteria:***

1. Population: Non-heteronormative individuals (LGBTQ+) within Pentecostal communities in South Africa.
2. Concepts: Studies addressing sexual identity, spiritual experiences, pastoral care, Pentecostal theology.
3. Context: South African Pentecostal churches, congregations, or literature.
4. Types of Sources: Peer-reviewed articles, theses, books, book chapters, reports, and grey literature.
5. Language: English.
6. Time Frame: 2015–2025.

***Exclusion criteria:***

1. Studies focusing exclusively on heterosexual populations.
2. Research conducted outside South Africa or outside Pentecostal contexts.
3. Studies not addressing sexuality, pastoral care, or theology.
4. Non-English publications.
5. Publications prior to 2015.

Justification: These criteria ensure the review is relevant to contemporary South African Pentecostalism, focuses on non-heteronormative experiences, and captures both academic and practical perspectives.

### Selection of sources

Titles and abstracts will be independently screened by two reviewers. Full-text articles meeting inclusion criteria will be retrieved and assessed. Discrepancies will be resolved through discussion or consultation with a third reviewer.

### Data extraction

A standardized charting form will capture:

• Author(s), year, publication type
• Study aims and objectives
• Population characteristics
• Methodology and study design
• Key findings related to sexual identity and Pentecostalism
• Theological perspectives and pastoral care approaches
• Research gaps

### Timeline

The scoping review will follow a structured schedule to ensure timely completion. The initial phase, including refining the research question, developing the search strategy, and piloting the search, will take 2 weeks. Database searches, retrieval of full-text articles, and screening of titles and abstracts will take 3 weeks. Full-text screening, data extraction, and charting will require 3 weeks, followed by thematic analysis, synthesis of findings, and drafting of the manuscript over 3 weeks. The final week will be dedicated to revisions, formatting, and preparation for submission. Overall, the project is expected to be completed within 3 months.

### Expected results

The scoping review is expected to systematically map literature on non-heteronormative sexual identities within South African Pentecostal communities, highlighting:

• The experiences of non-heteronormative individuals within Pentecostal contexts.
• Theological interpretations that influence inclusion, marginalization, or acceptance of sexual diversity.
• Documented pastoral care strategies supporting LGBTQ+ individuals.
• Psychosocial and spiritual outcomes of engaging with Pentecostal communities.
• Existing research gaps and opportunities to inform inclusive pastoral and theological practices.

The findings will provide a comprehensive overview of current knowledge, inform policy and faith-based interventions, and guide future research to promote acceptance, dignity, and spiritual well-being for non-heteronormative individuals in Pentecostal contexts.

### Ethical considerations

This study will analyse existing literature; no primary data collection is involved. Therefore, ethical approval is not required. Ethical conduct includes proper citation, acknowledgment of sources, and accurate representation of findings.

## Conclusion

This scoping review will synthesize available evidence on non-heteronormative identities within South African Pentecostalism, highlighting theological interpretations, pastoral care practices, and lived experiences. The findings will identify gaps in knowledge, inform inclusive pastoral and theological practices, and provide a foundation for future research that promotes acceptance, dignity, and spiritual well-being.

## Supplementary material

No supplementary materials are included at this protocol stage. Detailed search strategies, data extraction forms, and screening tools will be provided in the full scoping review upon completion.

## Data Availability

All data underlying the results are available as part of the article and no additional source data are required. Munyadziwa M. *SEXUAL TRAJECTORIES: Scoping Review Protocol.* OSF; 2025 Oct 7. Available from: https://doi.org/10.17605/OSF.IO/MYARU. ^23^ The dataset is licensed under a CC0 1.0 Universal license. The project contains the following extended data:
1. PRISMA-ScR Protocol Checklist
