# Supplementary material for: Sexual Trajectories of Non-heteronormative Identities in South African Pentecostalism: A Scoping Review Protocol
Source: F1000Res. 2025 Nov 4;14:1206. [Version 1] doi: 10.12688/f1000research.171581.1 (PMC12780513; doi:10.12688/f1000research.171581.1)
Supplement: Peer review history

# Reviewer report: Reviewer response for version 1

**DOI:** 10.5256/f1000research.189202.r443203

**Published:** 2026-01-09

**Authors:** José Barrera Blanco

**Affiliations:** 1 Universidad Complutense de Madrid, Madrid, Spain

This manuscript presents a scoping review protocol that aims to systematically map the literature on non-heteronormative identities within South African Pentecostal contexts. Drawing on Arksey and O’Malley (2005)’s scoping review framework, the study seeks to examine lived experiences, theological interpretations, and pastoral care practices affecting non-heteronormative individuals in Pentecostal communities. The protocol is clearly motivated, well situated within relevant theological and political debates, and addresses an important and underexplored phenomenon. Overall, the protocol demonstrates scholarly relevance and has the potential to make a meaningful contribution to the field of LGBTQ+ Religious Studies.

 The protocol is clearly written and structured, and the choice of a scoping review is appropriate and relevant given the lack of these type of studies in the field of LGBTQ+ Christianity. The aims and objectives are well articulated, and the rationale for conducting a scoping review is precise. However, I think that the study design is partly appropriate for the research question. This is not because a scoping review is an unsuitable approach, but rather because some elements of the research question, objectives and conceptual framing would benefit from further clarification to ensure a strong alignment between objectives, methods, and outcomes. Here are my suggestions and recommendations:

**1.   ** The research question and associated outcomes encompass a wide range of concepts, including psychosocial outcomes, inclusion, acceptance, and well-being. While these are important concerns, they may suggest an evaluative or outcome-focused synthesis that goes beyond what scoping reviews are typically designed. There is a risk of overstating what the review can meaningfully address.

**Recommendation:** The authors may wish to clarify that the review will map how issues such as inclusion, acceptance, psychosocial experiences, and well-being are discussed or represented in the literature, rather than attempting to assess their effectiveness or impact. Reframing the outcomes to focus on discourses and reported experiences within the PEO framework would bring the research question into closer alignment with a scoping review design.

**2.   ** The title introduces the notion of “sexual trajectories,” yet this concept is not explicitly defined or operationalised in the protocol. As it stands, it is unclear how studies on trajectories will be identified, particularly given the descriptive nature of scoping reviews.

**Recommendation: **Provide a clear definition/operationalization of what is meant by “sexual trajectories” in this study (identity development, disclosure, identity negotiation, inclusion/exclusion pathways, and their relation with religious belief and background).

**3.  **  The decision to limit the review to literature published between 2015 and 2025 would benefit from further justification. Given the longer history of debates around sexuality and Pentecostalism, earlier documents may still be highly relevant to the objectives of the review.

**Recommendation:** The authors could briefly justify the exclusion of literature published prior to 2015, acknowledge this as a limitation, or consider broadening the time frame to include earlier influential work. Any of these options would improve transparency and contextual depth.

Is the study design appropriate for the research question?

Partly

Is the rationale for, and objectives of, the study clearly described?

Yes

Are sufficient details of the methods provided to allow replication by others?

Yes

Are the datasets clearly presented in a useable and accessible format?

Not applicable

Reviewer Expertise:

I am a sociologist and social anthropologist specialising in the intersections of sexuality, religion, and politics. Over the past six years, I have researched the experiences of non-heterosexual Christians within institutional and church contexts, with particular attention to their collective action and strategies for challenging conservative discourses and practices. My work includes in-depth ethnographic research within LGBTQ+ Christian communities and organisations. As part of my doctoral thesis, I also conducted a scoping review of the literature on LGBTQ+ Christians from the 1970s onwards, which closely relates to the aims and methodological approach of this protocol.

I confirm that I have read this submission and believe that I have an appropriate level of expertise to confirm that it is of an acceptable scientific standard, however I have significant reservations, as outlined above.

# Reviewer report: Reviewer response for version 1

**DOI:** 10.5256/f1000research.189202.r443202

**Published:** 2026-01-07

**Authors:** Grant Nixon

**Affiliations:** 1 Sekolah Tinggi Teologi Ekumene (Jakarta Ecumenical Theological Seminary), North Jakarta, Indonesia

This article will explore various literatures addressing the experiences, theological interpretations, and pastoral care of non-heteronormative individuals among South African Pentecostals. Through an in-depth review of this literature, this study will present a discussion of the landscape over the past ten years in South African Pentecostalism.

 Therefore, this study has met academic standards by demonstrating clear, relevant objectives, research questions, and research methods, and—in my personal opinion—are crucial for the development of non-heteronormative studies, Christianity, and Pentecostalism.

 However, the question remains: how will all the collected literature be interpreted? What theoretical lens will be used? Furthermore, if this study aims to unravel the heteronormative perspective on theology and pastoral practice of South African Pentecostalism, how can a queer theological lens be integrated into the analysis and discussion of this article? All of this needs to be properly referenced to achieve the objectives of this study.

 Despite these concerns, this research holds great promise for untangling heteronormative bias in Pentecostal Christianity in South Africa in particular and other contexts that hold similar biases in general.

Is the study design appropriate for the research question?

Yes

Is the rationale for, and objectives of, the study clearly described?

Yes

Are sufficient details of the methods provided to allow replication by others?

Yes

Are the datasets clearly presented in a useable and accessible format?

Yes

Reviewer Expertise:

(Pentecostal) theology, gender, and sexuality studies

I confirm that I have read this submission and believe that I have an appropriate level of expertise to confirm that it is of an acceptable scientific standard.

# Reviewer report: Reviewer response for version 1

**DOI:** 10.5256/f1000research.189202.r439110

**Published:** 2026-01-02

**Authors:** Simon Wilhelm Kolbe

**Affiliations:** 1 SRH University of Applied Sciences, Fürth, Germany

The contribution presents a planned scoping review protocol that systematically examines the literature on experiences, theological discourses, and pastoral practices concerning non-heteronormative and sexually diverse individuals (LGBTQ+) within South African Pentecostalism. The aim is to consolidate existing knowledge, identify research gaps, and provide a foundation for the development of more inclusive theological and pastoral approaches.

 The planned study demonstrates a consistently well-prepared and well-balanced scientific approach, and the research idea is meaningful. This is because it is important not only for research, but also for religious, pastoral, social work, and political practice and research to understand how much knowledge exists on this relevant topic. The results are awaited with great interest. However, the derivations made by those conducting the study are particularly essential here, as what is implemented on the basis of the findings is what is truly relevant.

 Accordingly, throughout the data collection process, continuous recursive attention should be paid to clarity and to the precise definition and naming of the populations under study. The results must also be situated within a broader relational framework that takes the overall setting into account. In particular, confessional aspects of the respondents, as well as local differences in practice and geographical and demographic perspectives, would be of additional interest. A differentiation between positive and negative effects on individuals would likewise be valuable.

 Such a research project should therefore be positively approved; however, the researchers face a substantial responsibility to provide the scientific community with results that are not only analytically sound but also relationally grounded and applicable.

Is the study design appropriate for the research question?

Yes

Is the rationale for, and objectives of, the study clearly described?

Yes

Are sufficient details of the methods provided to allow replication by others?

Yes

Are the datasets clearly presented in a useable and accessible format?

Yes

Reviewer Expertise:

Social Work as a human rights profession; right-wing extremism and threats to democracy; political education and citizenship education; migration, forced displacement, and post-migrant societies; human trafficking and victim identification; prevention, intervention, and protection frameworks; professional theory and ethics of social work; digitalisation, technologisation, and future scenarios of social work; teaching, didactics, and Learning by Teaching (LdL); intersectionality, discrimination, and vulnerability; pastoral care, religiosity, and social work; transfer-oriented and applied research.

I confirm that I have read this submission and believe that I have an appropriate level of expertise to confirm that it is of an acceptable scientific standard.
